# Evaluation of Unowned Domestic Cat Management in the Urban Environment of Rome After 30 Years of Implementation of the No-Kill Policy (National and Regional Laws)

**DOI:** 10.3389/fvets.2019.00031

**Published:** 2019-02-19

**Authors:** Eugenia Natoli, Livia Malandrucco, Laura Minati, Stefania Verzichi, Raffaella Perino, Laura Longo, Francesca Pontecorvo, Anna Faini

**Affiliations:** Veterinary Service, Local Health Unit Rome 3, Rome, Italy

**Keywords:** unowned domestic cats, free-roaming domestic cats, control strategies, management, no-kill policy, Italy

## Abstract

Law no. 281, enacted by the Italian Parliament in 1991, was the first that aimed at managing urban free-roaming cats living in colonies, without killing and/or moving them from their site. It had been anticipated by the Lazio Regional Law no. 63/1988 and subsequently refined by the Lazio Regional Law no. 34/1997. These laws introduced: (i) the cats' right to live free and safe; (ii) the compulsory neutering of cats by the Veterinary Services of the Local Health Unit; (iii) the institutionalization of cat caretakers. Within this context, this paper intends to evaluate the effects of the application of the Italian laws on management of urban free-roaming cats for the years 1988 to 2018. To this end, some indicators have been built and applied to our activity data: number of censused colonies and number of cats; number of stable colonies due to neutering; number of hygiene and sanitary notifications; number of notifications to check cat welfare; number of bites by unowned free-roaming cats; number of notifications of cat poisoning. The number of citizens' requests for institutional interventions by public veterinary services in cat colonies management and, accordingly, the detection of cat colonies yet unknown, seem to confirm the interest of people to control the cat colonies in Rome in a humanitarian way, as evidenced in our data. This fact/phenomenon should be analyzed in its multiple dimensions, also including the many changes and social unrests which have affected the human-cat relationship in the last 30 years.

## Introduction

With regard to the evolutionary trend in global thinking and advocacy about unowned urban cat management, as Italian stakeholders, we were born on the right side of the world: in fact, it was back in 1991 that the Italian Parliament passed the first law (no. 281 /1991) that aimed at managing urban free-roaming cats living in colonies, without killing and/or moving them from their site. It had been anticipated by the Lazio Regional Law no. 63/1988 and subsequently refined by the Lazio Regional Law no. 34/1997. Thus, when Sheilah Robertson published her review (1), our 20 years of experience with TNR programs were an invaluable resource. The Italian National and Regional Laws introduced a revolutionary perspective which can be summed up in the following points: (i) the cats' right to live free and safe; (ii) the compulsory neutering of cats by the Veterinary Services of the Local Health Unit; (iii) the institutionalization of cat caretakers. The latter, gathered in Associations for Animal Protection or in Associations of Animalist Volunteers can, and should, be registered in a regional roll. Once registered, in agreement with the Public Veterinary Service and the Office for the Animal Welfare of the territory, they are officially assigned the management of a cat colony, but the mayor remains the responsible “owner” of the cats.

To verify the impact of the Italian TNR programs in reducing free-roaming cat populations in Rome, a survey was carried out in 2000 on 103 out of 965 cat colonies, where the cats had been previously neutered during the 1991–2000 sterilization campaign (2). The TNR programs resulted in a conspicuous decrease (16 to 32%) in total cat number, though not as great as expected. Furthermore, the effects were not felt until at least 3 years since neutering had passed, on account of the percentage of cat immigration due to abandonment and spontaneous arrival (ca. 16%). Thus, our results support Robertson (1) point of view stating that “….to have a large impact TNR will have to be adopted on a far greater scale than it is currently practiced” and, we would like to add, “it has to be matched with an effective educational campaign directed to citizens (that leads to responsible pet ownership) to reduce the high risk of owned-cat abandonment” (2). In Italy, sanitary education is enforced by law (National Law no. 833/1978) and is promoted in schools, during clinical activity in family counseling units and as part of the rabies prophylaxis after a bite from an animal. Furthermore, concerning all the activities for which we are responsible, the sanitary education is done by means of printed leaflets and informative material.

The intent of this paper is to evaluate, after 30 years, the effects of the application of the Italian laws on the management of urban free-roaming cats. Our hypothesis is that, in Italy, there has been an evolution in the human-cat relationship and, accordingly, we have built some indicators concerning not only cat demography control but also the emotional sensitivity to cat welfare.

## Definition of Feral Domestic Cats

Defining feral cats is still a complicated issue. It is assumed that all feral cats, no matter how they are defined, are not confined and roam freely, but there are still too many definitions based on different criteria: (i) origin (abandoned by humans, offspring of a feral female cat, lost by an owner); (ii) dependence on/independence from food supplied by human beings; (iii) socialization status to human beings (1, 3). To avoid further confusion, the term employed in this paper is “unowned” since, whatever the origin and the socialization level of cats (urban colony cats are a mixture of these categories), they do not have a single owner; according to the Italian laws the only owner responsible for urban feral cats is the mayor of the municipality.

## Materials and Methods

In order to evaluate the effects of 30 years of protectionist legislation, 7 indicators of our Local Health Unit activity have been constructed (Table 1). Activity data were collected by veterinarians working in our Unit, as the Italian law prescribes and, accordingly, they originate from our Unit database; every 3 months, data are to be transmitted to the Directorate-General of the Local Health Unit Rome 3 which, in turn, will forward them to the Regional Authority.

**Table 1:** Indicators utilized to evaluate unowned domestic cat management in the urban environment of Rome.

	**Indicator**	**No. of years**	**Site (see Figure 1)**
1	No. of censused colonies	30	Whole Rome/Local Health Unit Rome 3^*^
2	No. of cats	30	Whole Rome/Local Health Unit Rome 3^*^
3	No. of stable colonies due to neutering	30	Whole Rome/Local Health Unit Rome 3^*^
4	No. of hygiene and sanitary notifications	10	Local Health Unit Rome 3
5	No. of notifications to check cat welfare	10	Local Health Unit Rome 3
6	No. of bites by unowned cats	10	Whole Rome
7	No. of notifications of cat poisoning	10	Local Health Unit Rome 3

* *1988–2000 data collected from the whole city*.

**Figure 1:**
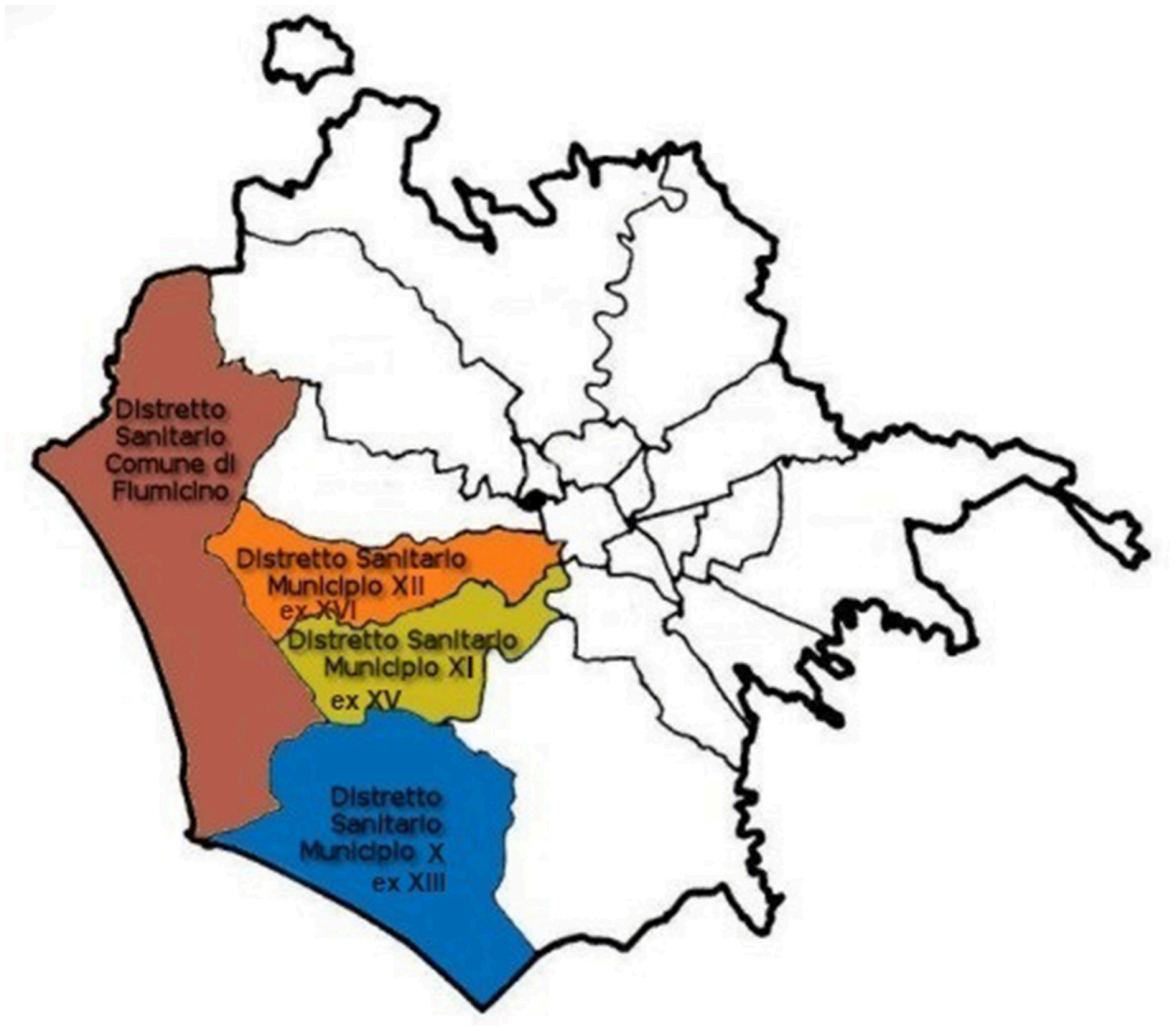
Map of Rome. Colored areas show the jurisdiction of Local Health Unit Rome 3.

The evaluation period ranges from 10 to 30 years (see Table 1); furthermore, depending on municipal ordinances, some indicators are applied to the whole territory of Rome whereas others are applied only to the area which is directly under the jurisdiction of our Local Health Unit (Figure 1).

Since 2008, the Ministerial Ordinance, and subsequent additions and modifications, has focused on animal poisoning. In the 10 years from 2008 to 2018, a database has been created on the various causes of death, with special attention to cases of poisoning. In fact, public and private veterinarians, police and private citizens can bring the dead bodies of animals and/or the suspected poisoned baits found in our territory (see Figure 1) for our Unit to make a diagnosis. All categories mentioned, i.e., public and private veterinarians, police and cat care takers that are private citizens, know the current laws; the latter are trained by the competent veterinary public service for the territory. Thus, most dead animals suspected of poisoning are brought to the dog shelter, also because citizens know that it is the collection center for dogs and cats found and collected dead on the street in the whole Rome.

Laboratory diagnoses were made by the Experimental Zoo-Prophylactic Institute of Latium and Tuscany Regions, in Rome, at the Chartered Institute which represents the National Reference Center for forensic veterinary medicine. The Institute routinely performs gross necropsy, histopathology, chemical testing and toxicology screening. The toxin tested is established based on the lesions found during autopsy. The following toxins are included: anticoagulants, pesticides, metaldehyde, strychnine, and zinc phosphide.

## Results

**Figure 2:**
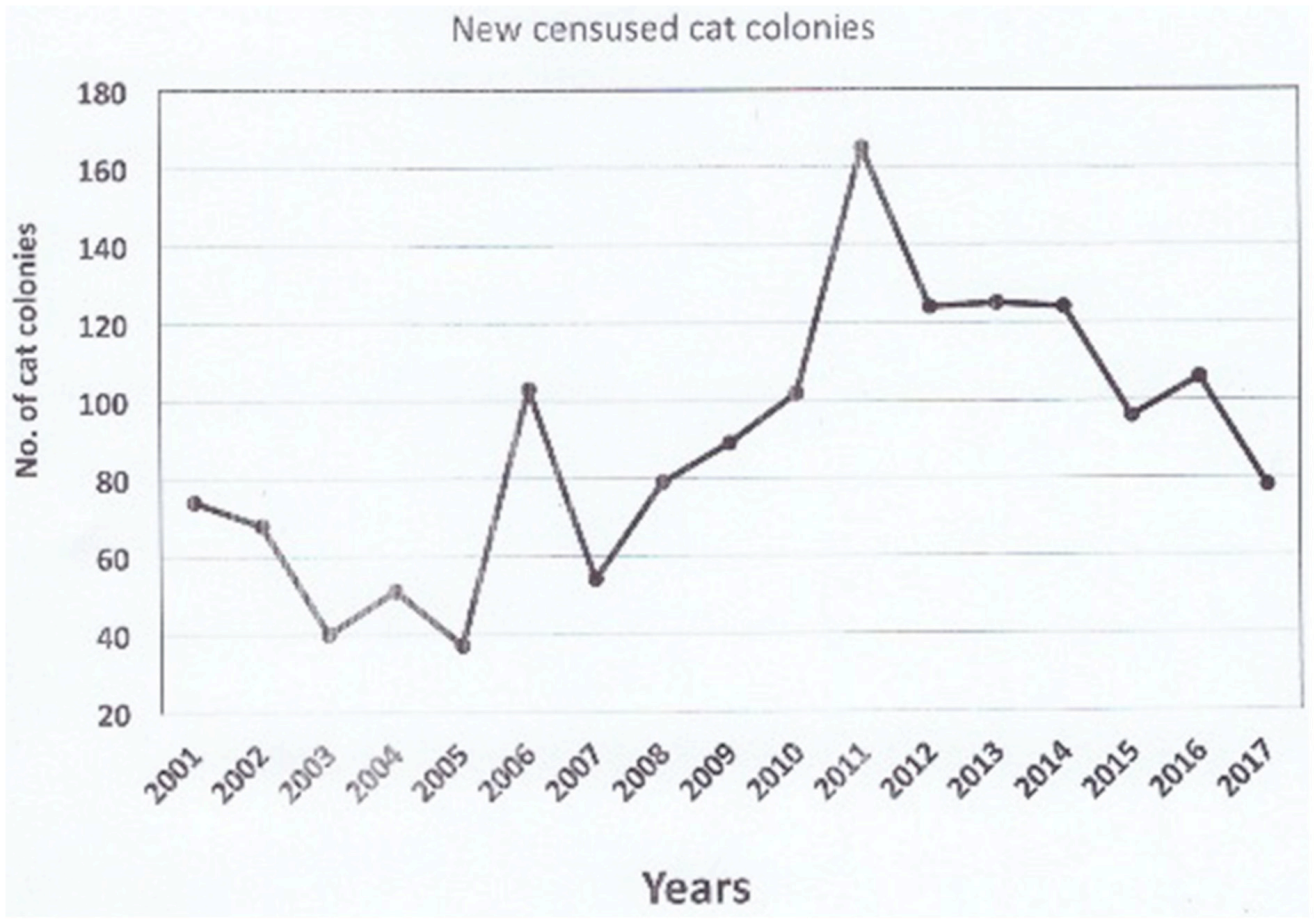
Yearly trend of new censused cat colonies.

**Figure 3:**
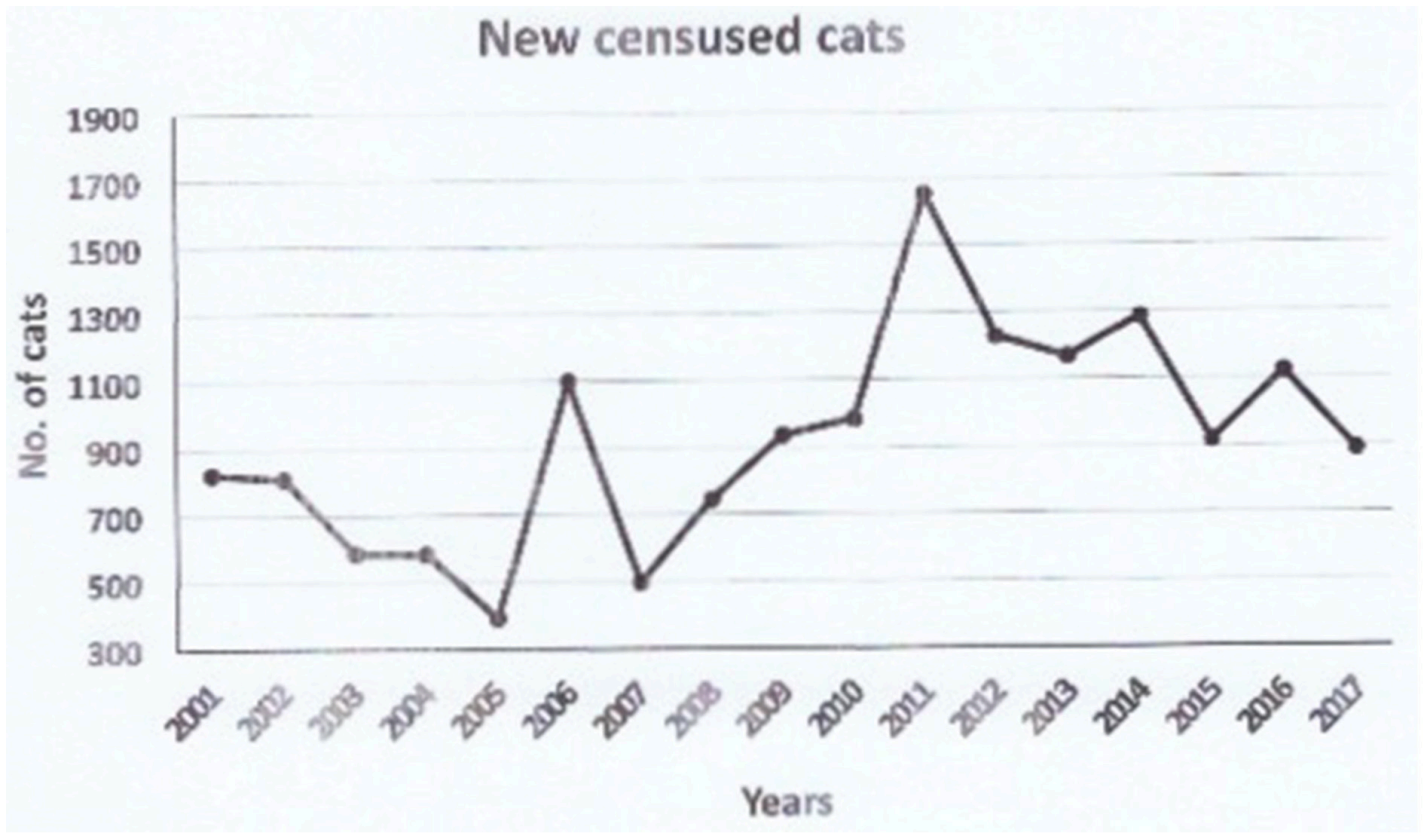
Yearly trend of new censused cats.

The total number of censused colonies composed of unowned cats (from 1988) was 1,878 in 2017, 89 of which have gone extinct. Since 2001, the number of new registered colonies of cats has increased, to reach a peak in 2011 (Figures 2, 3); after that year, the trend has started decreasing again. The total number of cats was 15,713, i.e., 8.37 cats per colony on average. Since the neutering campaign has begun, out of 1,878 cat colonies, 204 are stable thanks to the neutering.

Our Unit receives hygiene and sanitary notifications related to all animals in the urban environment, limited to the jurisdiction of Local Health Unit Rome 3 (see Figure 1). The notifications are mostly complaints about animal nuisance or about the control of their welfare. In the last 10 years, the total number of notifications was 1,002 (for dogs, pigeons, bats, parrots, owned cats, swallows, aquatic turtles), 84 of which concerned 84 different colony cats. Out of these 84 notifications received from citizens, 47 complained about hygienic and sanitary problems of the environment due to unowned cats, whereas 37 notifications requested control of cat welfare. The trend remained stable over the years.

Our Local Health Unit is also notified of any animal bite reports or claims filed for the whole city of Rome. In 10 years, out of 4,600, 143 were filed over domestic cat bites and only 6 over unowned cat bites.

Finally, in 10 years (2008–2017), we received 74 notifications of suspected cat poisoning, 21 of which were positive (proven by gross necropsy, histopathology, chemical tests and toxicology screening). The substances most commonly used were rodenticides (anticoagulants) (no. = 9), molluscicide (neurotoxic) (no. = 8) and a mixture of them (no. = 4). Since the above substances were found in edible baits near the dead body of the cats and in the dead cats' stomachs, it is highly probable that it was deliberate poisoning. In fact, the Italian laws forbid using these substances in public places; thus, if a poisoned meat ball is found in a public park, it is evident that it was deliberately put there to eliminate some animals.

The other causes of death were traffic accidents, predation by dogs and/or wild animals, infectious diseases and chronic silent diseases.

## Discussion

The first result reported here is the fact that in Rome the dynamic of cat populations has been monitored for 30 years, fulfilling the obligations of law, unlike many countries where unowned free-roaming urban cats are rarely quantified (4). Therefore, with the implementation of laws, the management of non-owned urban cats has become part of the profession of veterinarians, biologists, ethologists and operators of the National Health Service. This has lowered the share of emotionality that influences professional decisions. A positive consequence is that the debate on animal management is less emotional (both in the pro-cat and anti-cat sense) because the guidelines are established by law. Furthermore, management is financed entirely with public money.

The 2006 survey on 103 colonies of free-roaming unowned cats in Rome, based on the data gathered up to 2000 (2), yielded a 16–32% decrease in total cat number due to neutering, the positive effect of which was weakened by the percentage of cat abandonment and spontaneous arrival (around 16%). In the years 2000–2018, the TNR approach has been adopted on a greater scale (the whole city) and our Unit has never stopped matching it with an educational campaign for responsible pet ownership addressed to citizens (2). Have these actions yielded some changes? The results presented here suggest a positive trend with regards to the management of quantitative aspects. First of all, the yearly trend of new censused cat colonies, not only those identified by the Public Veterinary Service but, in most cases, those reported by citizens who submit a request for their authorized management, indicates that people in Rome are willing to look after unowned cats in a responsible way.

This latter issue is also confirmed by the peak of requests registered in 2011. A municipal project that started in 2010 took charge of the free neutering of owned cats. A by-result of this project was the increased promotion of the neutering of cat colonies: neutering has always been free, but not everyone was aware of this. As a consequence, in 2011 the number of requests for taking responsibility of cat colony management exploded, and only subsequently did the phenomenon normalize. No change in colony size was nonetheless registered.

Still, further evidence seems to support this general trend of interest in cat colony management. Despite the lack of precise data on abandonment (data are gathered and reported by volunteer cat caretakers, who are not professional operators), the undeniable fact is that 204 cat colonies are entirely composed of neutered cats and this makes them stable. Since some abandonment rate, although low, should be presumed along the years, the data seem to confirm the positive effect of the institutionalized management of cats by registered cat caretakers. In fact, few immigrated cats have replaced the dead or adopted cats. The demographic control put into action by TNR is the first step to a responsible management of unowned cat colonies. When a member of our research group (E.N.) started studying unowned free-roaming cat behavior in 1978, before the Italian laws on neutering were passed and no unowned cats were neutered yet, a slight constant increase in the number of cats was recorded throughout the years. The problem, however, was not only the increase in the number of cats, but rather the reason as to why such an increase was not as massive as expected: as a matter of fact, there was mass infant mortality [(5); p. 303]. About 90% of kittens living in the most “famous” and largest colonies in Rome died from various diseases, most often from rhinotracheitis (feline Herpes virus, Calicivirus). Kittens died with tangible and visible suffering. Thus, in terms of welfare, the TNR approach yields more benefits than costs.

Furthermore, if we consider the number of notifications from citizens asking for the control of unowned cats' welfare in the last 10 years (notifications were practically non-existent before 2008), there is evidence that Roman citizens are increasingly concerned for cat welfare. One of the concurrent causes of this trend could be the educational campaign on urban animals addressed to citizens.

Interestingly, in Rome, Toxoplasmosis sero-prevalence greatly decreased between 1991 and 2013. In fact, in 1990–1991 the sero-prevalence was 50.4% (IC 95% range 41–60%) (6), whereas in 2012–2013 it was 28% (IC 95% range 28–34%) (7).

The different rate of Toxoplasmosis sero-prevalence registered after about 2 decades is mainly attributable to the common practice of feeding cats (unowned and/or pets) with industrial food rather than with home leftovers and/or meat remnants from butchers. But data are still scarce, and more extensive studies will have to be carried out before formulating any conclusion.

The number of cat bite reports also deserves some comment: in 10 years, only 6 out of 4,600 reports were registered regarding non owned free-roaming cats. These numbers suggest why cats, unlike dogs, are not feared for their bites and aggressiveness. In fact, even when non owned, cats are not aggressive (with few exceptions) and people do not fear them. As the data discussed here have shown, the reports notified to our Unit from citizens complaining about hygienic and sanitary problems of the environment due to non owned cats, although still rare (on average 4.7 hygienic and sanitary notifications per year), were much more frequent than non owned cats bite reports (on average 0.6 bites per year).

Finally, although poisoning is not so frequent, it requires monitoring. Since 2008, thanks to the specific Ministerial ordinance enactment, and to other national laws against animal abuse, the attention of public bodies and private citizens has increased. This has resulted in a parallel increase in the number of reports filed. Of course, abuse and poisoning occurred also before 2008, but they were not notified. This is further evidence that institutionalized cat caretakers have become more sensitive and keener to know the causes of sudden cat deaths, mainly in order to prevent them and thus protect unowned free-roaming cats.

In conclusion, notwithstanding the fact that evidence from other parts of Italy point to still growing concerns for unowned free-roaming cat diffusion in terms of human health, animal welfare and social costs (4), in our opinion their management has greatly improved since 1988, and not only in Rome. Other big cities like Milan, Genoa and Florence have also attained valuable results [Genoa (9 cats/colony) and Florence (12 cats/colony) (8)], thanks to the efficient control activity put into action by the Public Veterinary Services. It would nonetheless be naïve to analyze the phenomenon without also accounting for the many other changes which have deeply affected human society in the last 30 years in Westernized Countries, including Italy. The constant registration of new cat colonies notified to our Unit does not necessarily imply a general increase in cat colonies. Often, in fact, people report or notify colonies which have long existed in the territory. Rather, this phenomenon probably testifies the increasing desire of people to manage them properly. Accordingly, more and more people ask for institutionalized support in cat colony management. This behavior could be determined by a variety of factors including, for instance, (i) the improvement, in the last 30 years, of the economic level and, accordingly, of the human welfare (thus making easy animal care); (ii) the increase of human sensitivity to the animal issue; (iii) the improvement of cat food quality (indirectly proven also by the decreased seroprevalence of toxoplasmosis); (iv) the wish of having more contact with nature and of relieving loneliness in a metropolis; (v) the increase of knowledge of the animal kingdom.

The results of this survey suggest that an evolution in the relationship between humans and cats has taken place in Italy, prompting the shift from demographic control to the adoption of a more sensitive attitude toward cat welfare.

### Laws

Lazio Regional Law no. 34 issued on 21/10/1997. Tutela degli animali di affezione e prevenzione del randagismo. Suppl. ord. no. 3, Bollettino ufficiale della Regione Lazio no. 30, 30 ottobre 1997.

Lazio Regional Law no. 63, issued on 09/09/1988. Istituzione anagrafe canina e protezione degli animali. GU 3^a^ Serie Speciale - Regioni n.36 del 09/09/1989.

Ministerial Ordinance 18th December 2008, and subsequent additions and modifications. Norme sul divieto di utilizzo e di detenzione di esche o di bocconi avvelenati. GU Serie Generale no.13 17/01/2009.

National Law no. 833, issued on 23/12/1978. Istituzione del Servizio Sanitario Nazionale. GU Serie Generale n. 360 del 28-12-1978–Suppl. Ordinario.

National Law no. 281, issued on 14/08/1991. Legge quadro in materia di animali di affezione e prevenzione del randagismo. Gazzetta Ufficiale no. 203, 30 August 1991.

National Law no. 189, come out 20/07/2004. Disposizioni concernenti il divieto di maltrattamento degli animali nonché di impiego degli stessi in combattimenti clandestini o competizioni non autorizzate. G.U. no. 178 of 31/07/2004.

## Ethics Statement

We did not need an institutional or governmental permission to carry on the study since it was an observational study and the neutering falls in good veterinary practice allowed by the National and International Laws.

Neither euthanasia, or any kind of animal sacrifice, was part of the study.

## Author Contributions

EN, LiM, and AF contributed conception and design of the study. LiM, LaM, SV, RP, LL, FP and AF organized the database. EN wrote the first draft of the manuscript. All authors contributed to manuscript revision, read and approved the submitted version.

### Conflict of Interest Statement

The authors declare that the research was conducted in the absence of any commercial or financial relationships that could be construed as a potential conflict of interest.
